# Transactions of the Buffalo Obstetrical Society

**Published:** 1886-04

**Authors:** 


### Transactions of the Buffalo Obstetrical Society.

*Stated Meeting, November 24, 1885.*

The President, Dr. W. W. Potter, in the chair; Dr. W. H. Thornton, Secretary.

Dr. M. Hartwig read a paper, entitled,

"HOW SHALL WE MANAGE CASES OF ABORTION?"

(See this Journal for January, 1886, for the full text of the paper.)

Dr. Wm. B. Hawkins coincided with the author in regard to the prophylactic effect of ergot in threatened abortion. He also believed in thoroughly emptying the uterus, either with the hand or forceps, in managing abortions; preferably with the hand, when possible, which was generally feasible where time enough was given to the manipulation. He also advocated antiseptic washings of the vagina and uterus after such manipulative procedures.

Dr. S. Y. Howell would simply emphasize a few of the statements made by the author of the paper. He recommended the introduction of a *small* tampon in the first threatenings as a prophylactic agent. The use of the colpenrynter was detrimental; so, too, was the thorough packing of the vagina in the Sims' position at this time. But it required, sometimes, a remarkably small amount of cotton to stop even a profuse hemorrhage in the early stages. Inasmuch as the prominent etiological factor in abortion was oftentimes syphilis, that fact should be borne in mind, and the fœtus carefully examined, noticing the ossific centers of the long bones, etc., that, if possible, future pregnancies might be brought to a successful issue.

Dr. R. L. Banta stated that, for the first five years of his practice, he invariably used the tampon when called to cases of abortion; then he began to use the forceps and curette, and although from this plan he had never yet lost a woman, he had seen resulting three or four severe attacks of pelvic cellulitis. He could not understand why a man should use a curette or forceps when the womb was sufficiently dilated to admit the passage of the finger. With one hand in the vagina, a finger in the uterus, and the other hand applied to the abdomen, he thought the uterus could nearly always be emptied. He would hesitate considerably before using a sharp curette. Regarding intra-uterine injections, in two cases where he had employed the sublimate solution, 1 to 4000, probably ten minutes after their use, he had seen, in one case convulsions, and in the other a marked chill follow, but without any deleterious results, and prompt recovery ensued in both cases.

Dr. Geo. E. Fell agreed with the essayist in that there was less danger of contaminating the uterus with septic matter by the use of the curette than with the finger. He thought, however, it always safe to employ antiseptic precautions after the employment of either.

Dr. W. D. Greene was impressed with the great variety of instruments employed to produce abortion. He had recently seen a case where a woman brought about this result by the use of a crochet hook; her temperature, on the second day, was 101° F, but she had no fever afterwards. He favored the use of the finger to empty the uterus any time up to three and a half months; with the sense of touch, we could better know where and what we were scraping. He did not use antiseptic intra-uterine washes as much as formerly, but thought they occupied a valuable place when properly employed.

Dr. Chas. G. Stockton favored the use of the finger rather than the curette for cleaning out the uterus; did not think one could tell as well, when using the curette, that no part of the placenta had been left behind; but with the finger, working gradually, it could nearly always be removed entirely. Thought that antiseptic washes often prevented any further trouble, even where some portions of the placenta were not brought away. He further believed that neither the tampon nor rubber bag should be employed where it was hoped to prevent abortion; that cases that are going to abort, will usually do so in spite of any and all precautions; and that rest, with the moderate use of opium, will do about all that can be done in the way of prevention. In a class of cases liable to abort from vascular and nervous excitement, the process might, oftentimes, be arrested by the judicious employment of anodynes. He related the history of an interesting case, in which abortion had occurred some ten or twelve times, but which ultimately gave birth to a living child, now two or three years old, and healthy; but the mother finally died, a year after her delivery, with diabetes mellitus.

Dr. P. W. Van Peyma agreed, in the main, with Drs. Banta, Greene and Stockton; had not much patience with "meddlesome midwifery," either at full term or in premature delivery; and regarded the curette as one of the new hobbies. In his practice, he gave ergot when the ovum did not come away, and he found this plan quite successful.

Dr. Milan Baker had never seen, in a quite extended experience, a fatal case of abortion; he had known of cases where the placenta had been carried two, two and a half, and even three months, with perfect recovery. In one case, where the placenta was carried two weeks, the woman was taken with severe hemorrhage, and he found her pale from the great loss of blood. The attending physician stated that the placenta came away with the ovum two weeks before. He found the placenta in the uterus, and removed it without difficulty, the woman making a complete recovery. *Per contra*, he was recently called in consultation where the attending physician had, eight days before, removed the fœtus, which he found lying in the vagina, and had filled the vagina, through a speculum, with pulverized alum. The woman died from septicæmia. The sum of all his experience was such as to teach him to wait long before resorting to instruments to remove the placenta, and to employ them only as a *dernier resort.*

Dr. Thomas Lothrop stated that, in the treatment of abortion, he was much of the opinion of Dr. Van Peyma, that meddlesome midwifery was a dangerous business. If we had retained secundines without fever, what harm did they do? In ninety-nine cases out of one hundred, there was no fever of significance. So, too, if there was no profuse hemorrhage, no harm came of waiting. He was, however, in favor of removing the placenta and secundines with the finger in all cases where it could be done easily. He had seen a case where the placenta was retained three months after the ovum was discharged, and he did not think himself justifiable in going into the uterus to find whether anything was wrong, when there was neither fever nor hemorrhage.

In the treatment of abortion, even if there was slight flowing, there was no possible need of the tampon; if the flowing was severe, then the tampon was demanded. If the pain was severe, control it with opium; if the flowing was profuse, control it with the tampon: these were the two prominent symptoms at the outset, and these were the methods of treatment. If, later, there were symptoms of septicæmia, then use thoroughly intra-uterus antiseptic washes. He regarded Dr. Hartwig's recommendations as decidedly radical and officious, but, nevertheless, very useful in dangerous cases.

The President regarded the paper as extremely interesting and instructive, in that it brought the subject down to the present date, expressing, as it did, the advanced views of some of the foremost men, particularly those of the German school. The feature that impressed him most, was that it treated of those cases where it was necessary to do something to save the life of the woman. He would criticise one expression which he had frequently heard use made of in the debate, "meddlesome midwifery." The term was often used with too much looseness. A man should be well enough equipped to know when to interfere, and when not to meddle; and this was about the sum of the whole business. Not to hold off our hands when there was necessity for action, could not be construed as meddlesomeness. He thought it would be well, in these discussions, to specify, with more definiteness, cases that might be let alone, and those that required treatment.

It was also important for us to distinctly understand what was meant by proper disinfection. It was very essential, when invading the uterine cavity, not only to wash the hands most thoroughly in antiseptic solutions, but also to dip a pine stick or match in the wash, and carry it well under the finger nails.

Two very prominent causes, inducing abortions, were retroversion of the womb and syphilis. Very many women habitually aborted from retroversion, and it was important, in such cases, to reposit the womb in the non-pregnant interval as a preventive measure. Syphilitic cases should also receive appropriate treatment, both during and preceding pregnancy. Much of the chronic anæmia and subinvolution which followed abortions could, he believed, be prevented by greater care in emptying the uterus, even in those cases where there were no marked immediate symptoms which seemed to urgently demand this practice; indeed, he could but regard these conditions as most generally due to failure in this particular; and would, on this account, urge upon the consideration of the members greater attention to removing the placenta and membranes, as a uniform practice in all cases.

Dr. Hartwig, in closing the discussion, said that it could not be denied that the finger had its scope and place in the management of these cases; but, neither could it be denied that there were some—nay, many—where little or nothing could be accomplished with it; and it was in these very cases where the scoop found its proper sphere. A chill would, truly, sometimes follow its use; so would it, likewise, follow the use of the antiseptic intra-uterine wash. But should we lay aside these valuable measures on this account? For himself he would answer the question negatively. Patients should, as a rule, be narcotized in curretting. He had never observed cellulitis to result from the measure.

